# Feasibility and implementation fidelity of a co-designed intervention to promote in-hospital mobility among older medical patients—the WALK-Copenhagen project (WALK-Cph)

**DOI:** 10.1186/s40814-022-01033-z

**Published:** 2022-04-09

**Authors:** Britt Stævnsbo Pedersen, Jeanette Wassar Kirk, Maren Kathrine Olesen, Birk Mygind Grønfeldt, Nina Thórný Stefánsdóttir, Rasmus Brødsgaard, Tine Tjørnhøj-Thomsen, Per Nilsen, Ove Andersen, Thomas Bandholm, Mette Merete Pedersen

**Affiliations:** 1 Department of Clinical Research, Copenhagen University Hospital Amager and Hvidovre, Kettegård Alle 30, Hvidovre, 2650 Copenhagen, Denmark; 2 Department of Endocrinology, Copenhagen University Hospital, Amager and Hvidovre, Denmark; 3 National Institute of Public Health, University of Southern Denmark, Copenhagen, Denmark; 4 Department of Medical and Health Sciences, Linköping University, Linköping, Sweden; 5 The Emergency Department, Copenhagen University Hospital, Amager and Hvidovre, Denmark; 6 Department of Clinical Medicine, University of Copenhagen, Copenhagen, Denmark; 7 Department of Orthopaedic Surgery, Copenhagen University Hospital, Amager and Hvidovre, Denmark; 8 Department of Physical and Occupational Therapy, Physical Medicine & Rehabilitation Research - Copenhagen (PMR-C), Copenhagen University Hospital, Amager and Hvidovre, Denmark

**Keywords:** Older medical patients, Mobility, Co-design, Accelerometers, Feasibility and fidelity, Implementation

## Abstract

**Background:**

Mobility interventions can prevent functional decline among older patients, but implementation of such interventions may be complicated by barriers in the clinical setting. The WALK-Copenhagen project (WALK-Cph) is aimed at promoting a 24-h mobility among older medical patients during hospitalization. The WALK-Cph intervention was co-designed by researchers and stakeholders to tailor the intervention to the clinical context. The aim of this study was to investigate the feasibility and implementation fidelity of the WALK-Cph intervention before evaluating clinical effectiveness in a randomized controlled trial (ClinicalTrials.gov NCT03825497).

**Methods:**

The WALK-Cph intervention consisted of six components: a welcome folder explaining the importance of in-hospital activity, a WALK-plan prescribing up to three daily walking sessions during and after hospitalization, a WALK-path in the hallway that patients were motivated to use daily, exercise posters in the hallways and bedrooms, self-service on beverages and clothes, and discharge with a WALK-plan. The present study reports on phase 2 of WALK-Cph and consists of a feasibility and a fidelity component. The study was conducted at the two WALK-Cph intervention departments after the initiation of the WALK-Cph intervention. A cohort of older medical patients (+65) was recruited for the feasibility study to assess recruitment and data collection procedures and the method for assessment of activity. Simultaneously, implementation fidelity was assessed by observing clinical practice and intervention delivery at the intervention departments.

**Results:**

A feasibility cohort of 48 patients was included. Inclusion was considered feasible with recruitment rates between 62% and 70% of all eligible patients. Also, data collection was conducted without obstacles, and all patients accepted to wear activity monitors. The fidelity observations showed that three of the six intervention components were partially implemented as planned whereas three components were not implemented as planned.

**Conclusion:**

The WALK-Cph intervention was found feasible, and although the intervention was not implemented with fidelity, the level of fidelity was considered sufficient to continue with further testing of the WALK-Cph intervention in a large-scale trial.

**Trial registration:**

ClinicalTrials.gov NCT03825497 (retrospectively registered). Protocol PubMed ID (PMID): 29523569.

**Supplementary Information:**

The online version contains supplementary material available at 10.1186/s40814-022-01033-z.

## Key messages regarding feasibility and fidelity

- We evaluated uncertainties in recruitment, data collection procedures, and the method for assessment of activity (i.e., *activ*PAL accelerometers) as well as the implementation fidelity of the intervention.
- The key feasibility and fidelity findings of the study are that data collection procedures were conducted without obstacles for all included patients, and all patients accepted to wear the *activ*PAL accelerometers. Regarding implementation fidelity, half of the intervention components, including the core components, were satisfactorily implemented.
- Based on the findings, it was decided to test the intervention in a large-scale trial.

## Background

Older adults who are hospitalized for medical illness often spend most of their time in bed being physically inactive [1–4]. This inactivity is associated with loss of independence, functional decline [2, 5–7], re-hospitalization, and death [2]. It appears that walking more than 900 steps per day during hospitalization is critical for the preservation of functioning [8]. Despite years of research, however, hospital-associated functional decline and mobility limitations remain a common challenge [9, 10].

Walking during hospitalization has proven effective in promoting mobility, reducing the length of stay and assuring discharge to own home [11]. Also, interventions promoting mobility among older adults during hospitalization [12–14], and exercise-based interventions initiated during hospitalization and continued after discharge [15], have shown promising results in preventing hospital-associated functional decline and in promoting functional ability [12, 13, 15], as well as in maintaining prehospitalization mobility [14]. However, perceived complexity (e.g., by health care professionals) of interventions and barriers in the local clinical context can complicate or hinder the implementation of such interventions [16, 17]. Several barriers to physical activity during hospitalization have been identified [16, 18–22]. These include lack of staff to assist with or encourage mobility [18, 19, 22], illness symptoms [18], fear of falls [18, 19], and a discouraging hospital environment [21].

To circumvent barriers to the implementation of mobility-promoting interventions, it has been suggested to engage stakeholders in the design of interventions [23] and to tailor the interventions to stakeholder perspectives and to the local clinical context [24, 25]. However, only a few studies have accounted for these recommendations [26–28] by adapting mobility interventions to the local context and assessing the effectiveness of these interventions. While studies of intervention efficacy in controlled settings may be useful for establishing the potential effectiveness of an intervention, fidelity of implementing such interventions in clinical settings can be challenged by external factors in hectic settings such as hospital wards [29]. Therefore, before undertaking large-scale evaluations of a co-designed complex intervention, it is recommended to investigate both the feasibility of the intervention (e.g., of the recruitment and assessment procedures) and the fidelity with which this intervention is implemented (i.e., if the intervention is delivered as intended) [29–31]. Investigating feasibility and implementation fidelity of an intervention can yield crucial insights into why a given intervention fails or works and how it may be improved [30].

To our knowledge, no previous study has investigated the feasibility and implementation fidelity of a mobility intervention in older medical inpatients before testing the effectiveness of the intervention. Therefore, the aim of this study was to investigate the feasibility and implementation fidelity of the WALK-Cph intervention before initiating a large-scale trial to investigate clinical effectiveness [32]. The WALK-Cph intervention was tailored to the local clinical context [25] and developed in a co-design process that involved stakeholders (i.e., health care professionals, patients and relatives) to facilitate implementation [33].

## Methods

As stated by Abbott JH [34], “a feasibility study is a preliminary study conducted specifically for the purpose of establishing whether or not a full trial will be feasible to conduct.” In this study, feasibility is based on the feasibility of recruitment and assessment procedures. Implementation fidelity is defined as “the degree to which teachers and other program providers implement programs as intended by the program developers” [35].

### Study setting and design

#### Study setting

WALK-Cph was carried out in Denmark, where a tax-funded healthcare system provides free treatment for all citizens for primary medical care, hospital care, and home-based care services. The present study was carried out at two departments (X and Y) located at two university hospitals (X and Y) in the Capital Region of Denmark. Patients admitted to the two departments are mostly acutely admitted older medical patients, and the two departments are similar in size and staff composition.

#### Intervention department at hospital X

Department X has 36 staff members consisting of nurses (*n* = 18), certified nursing assistants (*n* = 6), and physicians (*n* = 12). At hospital X, physiotherapists and occupational therapists are centrally organized in the Department of Physical and Occupational therapy and are therefore only present in the intervention department when they attend referred patients. The department has 24 beds for patients with endocrine disorders and is organized along two parallel hallways with bedrooms along the outer side of the hallways. In each hallway, there is a small, open common area (i.e., the hallway has double width for 6 m) with a table, a television and chairs. Beverages are available in the common areas in the hallways.

#### Intervention department at hospital Y

Department Y has 37 staff members consisting of nurses (*n* = 18), certified nursing assistants (*n* = 9), and physicians with responsibility in the department (*n* = 8). At hospital Y, physiotherapists and occupational therapists are a part of the multidisciplinary team in the intervention department, and they attend daily meetings where patient care plans are discussed. The department has 25 beds for patients with general medical disorders. The department is organized along two broad hallways with bedrooms on one side. In each hallway, there is a common room where patients can eat, watch television, and talk to other patients and relatives. Beverages are available in the common room and in the hallways.

### Study design

WALK-Cph [36] is a multidisciplinary and cross-sectoral project that aims to promote increased daily mobility among older medical patients (+65) during and after hospitalization. The project is inspired by a hybrid 2 design [37, 38] with parallel testing of an intervention to promote 24-h mobility and a plan for implementation of the intervention. It is organized in four phases combining quantitative and qualitative methods (Table 1).

**Table 1:** WALK-Cph timeline

Phases	Time point	Action taken		
**1**	**Sept 2016**	***Randomization*** 2 intervention departments 2 control departments		
	**Nov 2016–May 2017**	***Baseline cohort*** Assessment of daily mobility in older medical patients at all departments	***Baseline field study*** Observation of daily practice in all departments	
	**March 2017–Sept 2018**	***Co-design of intervention (researchers and stakeholders)*** Workshops to design intervention	***Co-design of implementation plan (researchers and stakeholders)*** Workshops to design implementation plan	***Barrier screening physicians*** Barrier screening interviews with physicians
**2**	**Sept 2018**^**X**^ **Feb 2019**^**Y**^	***Implementation of intervention*** Initiated at the two intervention departments		
	**Sept–Nov 2018**^**X**^ **Feb–March 2019**^**Y**^	***Feasibility study (I + II)*** Feasibility of recruitment procedures, assessment procedures, method for assessment of daily mobility	***Fidelity study (I + II)*** Observation of daily practice related to WALK-Cph intervention components	
	**Dec 2018**^**X**^	***Re-design of intervention (researchers and stakeholders)***^***a***^ Workshops for refinement of intervention based on results from feasibility and fidelity studies	***Re-design of implementation plan (researchers and stakeholders)***^***a***^ Workshop for refinement of implementation plan based on results from fidelity study	
	**Jan–Feb 2019**^**X**^	***Feasibility study (III)*** Feasibility of recruitment procedures, assessment procedures, method for assessment of daily mobility	***Fidelity study (III)*** Observation of daily practice related to WALK-Cph intervention components	***Questionnaires***^***a***^ Stakeholder view on use of implementation plan and implementation of WALK-Cph intervention components
	**Feb–April 2019**^**a**^	***Semi-structured interviews with patients*** Semi-structured interviews with patients in the WALK-Cph intervention (20 partients)		
**3**	**March–Dec 2019**	***Effect of intervention*** Randomized controlled trial^b^	***Implementation of intervention*** Observational study	
**4**	**Feb 2020**	***Adoption*** Observations of daily practice Semi-structured interviews		

*X* department X, *Y* department Y

^a^This part of phase 2 is not reported on in the present study

^b^Changed to cohort study due to close down of one of the two intervention departments

The design of the WALK-Cph project has been described by Kirk et al. in the study protocol [36]. Briefly, to provide some context, in phase 1, four medical departments at hospitals in the Capital Region of Denmark were included in WALK-Cph and block-randomized to two intervention and two control departments. A baseline cohort study was performed to collect data on daily mobility among older medical patients hospitalized at the four departments, and a baseline field study was performed to investigate the social context and practice related to patients’ mobility in the four departments [16]. Hereafter, at co-design workshops, stakeholders (health care professionals, patients, relatives, and researchers) from the two intervention departments (Departments X and Y) collaborated on designing the WALK-Cph intervention and the WALK-Cph implementation plan. Subsequently, implementation of the WALK-Cph intervention was initiated in Departments X and Y and the feasibility and implementation fidelity of the intervention were evaluated simultaneously (Table 1, phase 2). The present study reports on data from both components of this phase concerning trial procedure feasibility [34, 39, 40] and implementation fidelity of the WALK-Cph intervention in the two intervention departments, to decide on the suitability of the intervention for effectiveness testing (phase 3). Also, data from the baseline studies will be presented for comparison. The two components, which constitute the present study, will be referred to as “the feasibility study” and “the fidelity study” throughout. For more information on the process, please see Kirk et al. [36].

#### The WALK-Cph intervention

The WALK-Cph intervention consists of the following six components: (1) a welcome folder with a paragraph focusing on the importance of being physically active during hospitalization. The folder is handed out to patients on admission, (2) a WALK-plan encouraging three daily walking sessions. The WALK-plan is prescribed by a physician and handed out to patients by either nurses or physiotherapists, (3) a WALK-path, marked in the hallway. All health care professionals encourage patients to walk along the path once daily, (4) posters with three simple exercises that all health care professionals encourage patients to carry out. The posters are placed in the hallways and bedrooms, (5) self-service on beverages (Department X & Y), food (Department Y), and clothes (Department X), which is encouraged by all health care professionals, and (6) discharge with a WALK-plan and follow-up by the municipality (For further details, please see Additional file 1).

#### Patient recruitment

Patients were recruited for the feasibility study during three feasibility periods (feasibility periods I, II, and III) (Table 1). Each feasibility period lasted 4 weeks. Feasibility period I was conducted at the intervention department at hospital X. Based on the results from feasibility period I, adjustments were made to the intervention and the implementation plan and hereafter feasibility period II was conducted at the department. Feasibility period III was like feasibility period I and was conducted at the intervention department at hospital Y. A second feasibility period was not carried out at hospital Y since a shutdown of the department was announced by May 2019 as part of a restructuring and reorganization of the hospitals within The Capital Region of Denmark.

### Feasibility and fidelity studies

#### The feasibility study

The feasibility study was a preliminary study [34, 39, 40], which was conducted at the two intervention departments after the initiation of the WALK-Cph intervention. The aim of the study was to investigate whether it would be feasible to conduct a full trial for the assessment of intervention effectiveness. Thus, we evaluated the feasibility of the WALK-Cph intervention procedures before continuing to phase 3 of WALK-Cph (Table 1). Therefore, no effective testing was made, and no distinction was made between primary and secondary outcomes [34]. The study was performed from September 2018 to March 2019 (Table 1).

##### Participants and recruitment

We included older medical patients (+65) admitted from their home to one of the two intervention departments. Patients were excluded on the following criteria: inability to walk independently with or without walking aids, inability to speak and understand Danish, inability to cooperate, transferal to another department or another hospital, terminal illness, ongoing cancer treatment, isolation room-stay, or referral to nursery home. During all three feasibility periods, patients were recruited on all weekdays. Recruitment of patients was performed by two investigators from the research team (BSP and MMP) and two research assistants (BJ and RB), who also undertook inclusion during phase 3. Through a daily review of medical records, eligible patients were identified and hereafter contacted by one of the investigators. Patients who gave oral and written informed consent underwent an interview and mobility assessments.

##### Outcomes

The assessed outcomes were feasibility of recruitment procedure (percentage of eligible patients willing to participate), the feasibility of assessment procedures (percentage able to participate in assessments), the feasibility of the method for assessment of primary outcome for randomized controlled trial (percentage of included patients willing to wear *activ*PAL3^TM^ accelerometers), and level and variability in 24-h mobility. Based on a previous feasibility study in our hospital [41], we applied the following criteria for feasibility: (1) 50% of all eligible patients should participate and (2) all included patients except a maximum of 2 patients should participate in the assessments and wear the accelerometers.

##### Data collection

At inclusion, all included patients underwent a structured interview to collect information on place of residence, marital status, level of education, pain, use of municipal help, walking aids, number of falls within the past 12 months, level of physical activity (PA), mobility status, activities of daily living (ADL), and cognitive status. Also, daily mobility, the primary outcome for the following randomized controlled trial (phase 3), was assessed. The “Saltin-Grimby Physical Activity level Scale” [42–44] was used to assess the level of physical activity, the De Morton Mobility Index (DEMMI) [45, 46] and The Life-Space Assessment (LSA) [47–49] to measure mobility status, the Barthel Index 20 [50] to quantify ADL, and the Short Orientation-Memory-Concentration test (OMC) [51] to assess cognitive status. The *activ*PAL3^TM^ accelerometer was used to assess mobility through the following outcomes: uptime (i.e., time spent walking and standing), time spent lying, and number of steps. All assessments were performed on admission and the *activ*PAL3^TM^ activity monitor was worn for the following 48 h during hospitalization. The *activ*PAL3^TM^ is valid and reliable in measuring postures in mobility-impaired older people [52, 53] and in measuring walking at gait speeds between 0.67 m/s and 1.56 m/s [54, 55].

##### Sample size

No formal sample size estimation was made since this was a feasibility study where no effectiveness testing was planned and performed [34, 39]. Approaches to sample size justification for pilot and feasibility trials vary. We aimed for a target sample size of 16 patients for each feasibility period (taking a dropout rate of 25% into account), based on Julious [56], who recommends 12 participants per group as a rule of thumb for pilot studies.

##### Data analysis

Recruitment rates (i.e., percentage of eligible patients willing to participate), patient characteristics, and results on 24-h mobility are presented as descriptive data given as medians with interquartile ranges, means with standard deviations or percentages depending on variable types. Recruitment rates, patient characteristics, and results on 24-h mobility are presented separately for each department and feasibility period. SAS Enterprise Guide 7.1 (SAS Institute Inc., Cary, NC, USA) was used for all statistical analyses, and all analyses were performed by BSP and MMP. The reporting of this study follows the Consolidated Standards of Reporting Trials (CONSORT) extension for pilot and feasibility trials with simple adaptations as recommended by Lancaster and Thabane [57].

#### The fidelity study

##### Procedures

To examine the implementation fidelity of the WALK-Cph intervention, a fidelity study performed as an ethnographic study [58] was carried out at the two intervention departments at the same time as the feasibility study, from September 2018 to March 2019 (Table 1). The study was carried out as participant observations of daily practice at the two intervention departments [59]. The observations focused on gathering information about the daily practice concerning the WALK-Cph intervention components that were related to hospitalization (Additional file 1). No observational data were collected on WALK-Cph intervention components to be performed after discharge (i.e., WALK-plan after discharge). Information on this component was collected through patient records.

##### Data collection

Data were collected through observations conducted at the two intervention departments during the feasibility periods using a focused observation strategy [60]. The observations followed an observation guide (Table 2) inspired by Hasson’s [61, 62] modification of Carroll’s Conceptual Framework for Implementation Fidelity [63] and was based on the components of the WALK-Cph intervention. All observers were instructed to follow the observation guide and be especially observant towards components of the framework that could be evaluated through observations: adherence (content, frequency, dose), quality of delivery, participant responsiveness, and context [61–63]. Adherence was defined as “how far those responsible for delivering an intervention actually adhere to the intervention as it is outlined by its designers” [63] and related to the delivery of the intended content and dose of the intervention [62, 63]. According to Carroll, adherence is the essential component of implementation fidelity. Obtainment of full adherence can be moderated by factors such as the quality of delivery, participants’ responsiveness [62, 63], and context [61]. The quality of delivery “concerns whether an intervention is delivered in a way appropriate to achieving what was intended” [63] and relates to the quality in delivering the intervention components [62]. Participant responsiveness refers to the engagement of those responsible for delivering the intervention and how they perceive the relevance of the intervention [62, 63]. Context refers to “factors at political, economical, organizational, and workgroup levels that affect the implementation” [62]. The observers could note observations that were not related to the components in the guide if something was deemed interesting or relevant. Observations were carried out as participant observations of the work practices in the departments. Thus, all involved staff and hospitalized patients could be observed as well as their mutual interaction, as this was expected to affect the delivery of the intervention. For example, such an interaction was observed when a health care assistant told a physician, “we need to give a WALK-plan to patient X.” The observer noted how staff and patients acted in relation to the different intervention components. Observations were carried out on weekdays and covered both day and evening shifts in a randomized order. Each session lasted 2–4 h and consisted of the following staff around the department, following physicians around the department, observing in the hallway, and observing in the staff office. All observers were instructed to ask for permission before following a given health care professional and to leave a room or situation if staying felt disturbing or unethical. During the three periods, observations were performed by BSP, JWK, BMG, NTS, RB, MMP, and three research assistants (5 physiotherapists, 2 nurses, 1 medical student, and 1 anthropologist). We chose to use observers with different professional backgrounds to add multidisciplinary breadth to the observations [64]. We used several observers because focused observation can be exhausting and requires full concentration. Field notes were written during and immediately after the observations.

**Table 2:** In-hospital observation guide for observers

In-hospital intervention components	Observations
Welcome folder	Do health care staff hand out welcome folders to patients on admission? (S) Is the folder introduced with a focus on mobility during and after hospitalization and by whom?
WALK-plan	Do health care staff/physicians contact patients regarding WALK-plan and physical activity during hospitalization? (S/P) Are WALK-plans handed out to patients? By whom? (S/P) During rounds, do health care staff talk to the patients regarding WALK-plans? (S) Do patients follow their WALK-plans? (H) Who attend board meeting at 1 pm? (S/P) Are WALK-plans mentioned at the board meetings? (P) At board meetings, do physicians inform about the WALK-Cph intervention and do they follow up on prescriptions? (P) Do physicians express challenges regarding prescription of WALK-plans? (P) Do those responsible for implementation of the intervention mention the intervention at board meetings? (P)
WALK-path	Do health care staff/physiotherapists contact patients regarding use of the WALK-path? (S) How are patients motivated and by whom? Are patients introduced to the WALK-path and the exercises? (S) How are patients introduced and by whom? Do patients go to the WALK-path/Are patients accompanied to the WALK-path? (S/H) How are patients accompanied and by whom? Do patients exercise independently by the WALK-path? (H) If they don’t, why not?
Posters with exercises	Do health care staff contact patients regarding use of exercises on posters? (S) How are the patients motivated and by whom? Do the patients exercise guided by the posters? (S)
Self-service on clothes^a^	On admission, do health care staff introduce patients to the wardrobes and self-service on clothes on admission and do they show the patients the location of the wardrobes? (S) Do health care staff contact patients regarding self-service on clothes? (S) How are the patients motivated and by whom? Do patients collect clothes independently/are patients assisted to collect clothes? (S/H) Who assist patients and how?
Self-service on beverages	On admission, do health care staff introduce patients to self-service on beverages in refrigerator and by beverage cart and do they show patients the location of the refrigerator / beverage cart? (S) Do health care staff contact patients regarding self-service on beverages? (S) How are the patients motivated and by whom? Do patients collect beverages independently? (H) If patients are assisted, who assists?
Discharge with a WALK-plan	*This component was not observed but assessed via patient records*

^a^This component was not a part of the intervention in department Y; *(S)* observer follows staff, *(H)* observer sits in hallway, *(P)* observer follows physician, and *(O)* observer is in staff office

##### Data analysis

Field notes from the fidelity study consisted of 222 pages from the three fidelity periods. The fidelity analysis was performed by BSP, JWK, and BMG. The field notes were read, re-read, and coded in a deductive process structured after Hasson’s [62] modified version of The Conceptual Framework for Implementation Fidelity [63]. All field notes were read individually by all three authors, and the text was coded according to the fidelity components (i.e., adherence, quality of delivery, participant responsiveness, and context). Hereafter, the codes were discussed between the authors until agreement on coding was obtained. For example, when WALK-plans and level of WALK-plans were discussed between different members of the staff, this was coded as “adherence.” After ended coding, five authors (BSP, JWK, RB, NTS, and MMP) carried out consensus discussions to determine an estimated level of fidelity for each of the intervention components. This level of fidelity was based on the five authors’ estimation of the degree of delivery based on the adherence component [63] (content, frequency, dose) throughout all observations: (1) “not delivered as planned,” if the three authors agreed that the overall delivery of the component was considered at the <30% level of the intended delivery (e.g., if two observations show patients collecting clothes and 8 observations show staff collecting clothes); (2) “partly delivered as planned,” if the three authors agreed that the overall delivery of the component was considered at the 30–60% level of the intended delivery; (3) “delivered as planned,” if the three authors agreed that the overall delivery of the component was considered at the >60% level of the intended delivery.

As part of co-designing the WALK-Cph intervention, the research team scored the feasibility of all possible intervention components [36] informed by the Delphi method [65] and rated each component on its ability to enhance the likelihood of mobility on a 1 (yes) to 5 (no) scale. The WALK-plan and the WALK-path were rated as highly able to enhance mobility (score of 1), the welcome folder as moderately able to enhance mobility (score of 2) and the posters, and the self-service and discharge with a WALK-plan as neutral (score of 3). Based on these ratings, the WALK-plan and the WALK-path were considered core components. Therefore, it was decided that these two components needed to be at least partly delivered as planned during phase 2 for the intervention to be sufficiently implemented to be carried forward. This was a pragmatic choice based on an awareness that full implementation cannot be obtained within a month, but likely requires 6 to 12 months [66] and that fidelity of complex interventions is not straightforward and may change over time [30].

## Results

### Feasibility study

#### Recruitment of patients

**Fig. 1:**
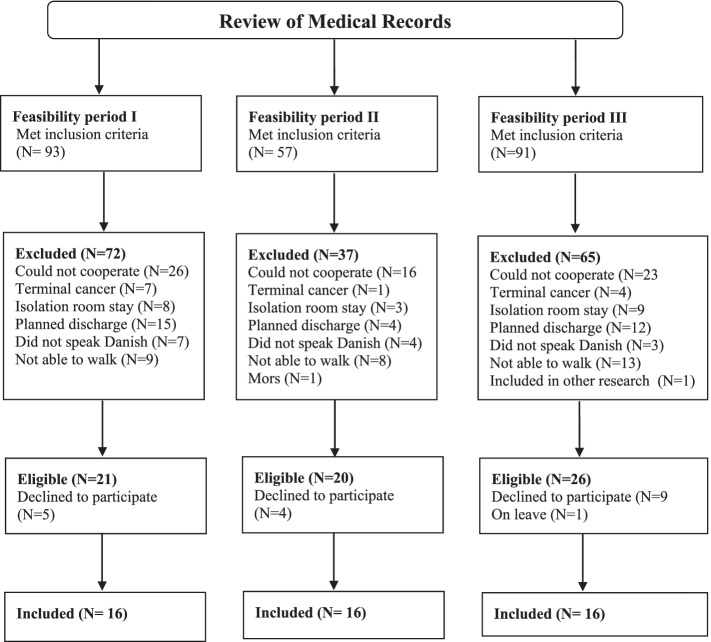
Flow of patients in a feasibility cohort

For a visual presentation of the flow of patients throughout the study, see Fig. 1.

After a review of medical records, a total of 241 patients met the inclusion criteria in feasibility period I (*N*=93), II (*N*=57), and III (*N*=91). Of those, 174 patients were excluded (*N*=72 in feasibility period I, *N*=37 in period II, and *N=*65 in period III). Reasons for exclusion of patients were 65 patients could not cooperate in assessments, in 31 patients discharge was already planned, 30 patients could not walk independently, 20 patients were in isolation and were therefore not allowed to leave their room and engage in the intervention components, 14 patients did not understand or speak Danish, 12 patients had terminal cancer,1 patient died before assessments, and 1 patient participated in another research project. In total, 67 patients were eligible according to the in- and exclusion criteria. Of those, 18 declined to participate and 1 was on leave. Therefore, 48 patients (70%) were included in the feasibility study (16 in each feasibility period). Recruitment rates for feasibility periods I, II, and III were 70%, 70%, and 62%, respectively. The characteristics of patients included in the feasibility study are presented in Table 3. Overall, the patients were 77.4 years old (SD 7.6), 44% were female, 65% lived alone, 96% lived in their own home, 65% had municipal help, and 65% used walking aids.

**Table 3:** Characteristics of feasibility study participants

	Hospital A						Hospital B			
	Baseline cohort		Feasibility cohort I		Feasibility cohort II		Baseline cohort		Feasibility cohort III	
	*N*		*N*		*N*		*N*		*N*	
Age; mean (SD)	20	80.2 (6.1)	16	78.4 (9.0)	16	76 (7.2)	20	78.1 (7.5)	16	77.8 (6.7)
Gender, female; *n* (%)	20	14 (70%)	16	7 (44%)	16	7 (44%)	20	15 (75%)	16	7 (44%)
Living alone, yes; *n* (%)	20	3 (15%)	16	10 (63%)	16	11 (69%)	20	14 (70%)	16	10 (63%)
Marital status, *n* (%)	20		16		16		20		16	
Married		4 (20%)		8 (50%)		7 (44%)		6 (30%)		4 (25%)
Widow(er)		9 (45%)		4 (25%)		2 (13%)		3 (15%)		7 (44%)
Divorcé(e)		5 (25%)		2 (13%)		4 (25%)		6 (30%)		4 (25%)
Not married		2 (10%)		2 (13%)		3 (19%)		5 (25%)		1 (6%)
Education, *n* (%)	17		15		16		20		12	
<High school		3 (18%)		8 (53%)		3 (19%)		1 (5%)		1 (8%)
High school		2 (12%)		1 (7%)		0		0		0
Skilled		10 (59%)		0		8 (50%)		7 (35%)		1 (8%)
Graduate		2 (12%)		4 (27%)		2 (13%)		9 (45%)		7 (58%)
Postgraduate		0 (0%)		2 (13%)		3 (19%)		3 (15%)		3 (25%)
Residence, *n* (%)	20		16		16		20		16	
Own home		16 (80%)		14 (88%)		16 (100%)		19 (95%)		16 (100%)
Intermediate care facility		0		0		0		0		0
Senior housing		4 (20%)		2 (13%)		0		1 (5%)		0
With family		0		0		0		0		0
Use of walking aids, yes; *n* (%)		14 (70%)		13 (81%)		10 (63%)		8 (40%)		8 (50%)
Municipal help, yes; *n* (%)	20	19 (95%)	16	9 (56%)	16	9 (56%)	20	12 (60%)		13 (81%)
Personal help, yes; *n* (%)		8 (40%)		5 (31%)		1 (8%)		3 (15%)		1 (6%)
Falls (last 12 months), yes; *n* (%)	20	10 (50 %)	16	12 (75%)	16	10 (63%)	20	9 (45%)	16	6 (38%)
1 time		3 (30%)		6 (50%)		8 (50%)		3 (33%)		1 (17%)
2 times		1 (10%)		3 (25%)		2 (13%)		2 (22%)		3 (50%)
3 times		2 (20%)		1 (8%)		0		1 (11%)		0
4 times or more		4 (40%)		2 (17%)		0		3 (33%)		2 (33%)
Physical activity level (PA), *n* (%)	20		16		16		20		16	
Low PA		12 (60%)		12 (75%)		3 (19%)		4 (20%)		4 (25%)
Moderate PA		8 (40%)		4 (25%)		13 (81%)		12 (60%)		12 (75%)
High PA		0		0		0		4 (20%)		0
Barthel20, mean (SD)	20	15.6 (3.6)	16	16.3 (3.7)	15	19 (1.7)	20	16.7 (3.6)	16	18.1 (2.0)
OMC, mean (SD)	19	18.3 (6.6)	16	20 (5.2)	16	21.4 (4.1)	20	24 (4.8)	16	21.9 (5.7)
DEMMI, mean (SD)	20	50.6 (18.6)	16	60.4(18.5)	16	67.4 (12.6)	20	61.7 (29.2)	16	68.3 (20.0)
LSA, mean (SD)	20	38 (26,1)	16	41 (21.5)	16	65.8 (23.8)	20	73 (37.3)	16	50.6 (26.8)
Pain, yes; *n* (%)	20	5 (25%)	16	11 (69%)	16	9 (56%)	20	6 (30%)	16	9 (56%)

*OMC* Orientation Memory Concentration Test, *DEMMI* De Morton Mobility Score, *LSA* The University of Alabama at Birmingham (UAB) Study of Aging Life-Space Assessment (LSA)

#### Assessments

All patients, who were included in the feasibility study, completed the structured interview and underwent assessments. However, one patient from feasibility period I and six patients from feasibility period III did not wish to inform about their level of education, and for one patient in feasibility period II, data on one item of the Barthel-20 were missing. All included patients accepted to wear the *Activ*Pal3^TM^ accelerometer. Results on 24-h mobility among patients included in the feasibility study during the three feasibility periods are presented in Table 4. The median uptime (i.e., time spent standing and walking) for patients included in the feasibility study was 2.11 h/day (IQR 1.48;3.04), 1.63 h/day (IQR 0.65;2.86), and 2.05 h/day (IQR 1.12;3.57) for feasibility periods I, II, and III, respectively. The median time spent lying in bed was 21.3 h/day (IQR 20.67;22.44), 21.6 h/day (IQR 20.4;23.3), and 21.7 h/day (IQR 20.44;22.38) for feasibility periods I, II, and III, respectively. The median number of steps taken was 1055 steps/day (IQR 308;1953), 678 steps/day (IQR 197;2128), and 1893 steps/day (IQR 1339;3324) for feasibility periods I, II, and III, respectively.

**Table 4:** A 24-h mobility assessed over 48 h in feasibility cohorts

**Hospital A**			
24 h	Baseline cohort *N*=20, median (IQR)	Feasibility cohort I *N*=16, median (IQR)	Feasibility cohort II *N*=16, median (IQR)
Uptime, h/day	1.26 (0.69, 2.12)	2.11 (1.48, 3.04)	1.64 (0.65, 2.86)
Number of steps, no./day	518 (115, 1333)	1055 (308, 1953)	678 (197, 2128)
Time spent lying, h/day	22.7 (21.89, 23.29)	21.3 (20.67, 22.44)	21.6 (20.4, 23.3)
**Hospital B**			
24 h	Baseline cohort *N*=20, median (IQR)	Feasibility cohort III *N*=16, median (IQR)	
Uptime, h/day	1.98 (1.02, 3.28)	2.05 (1.12, 3.57)	
Number of steps, no./day	1150 (486, 2236)	1893 (1339, 3324)	
Time spent lying, h/day	21.4 (20.65, 22.47)	21.7 (20.44, 22.38)	

### The fidelity study

#### Fidelity analysis

Results from the fidelity analysis are presented in Table 5. The observations showed that none of the intervention components were delivered as planned. Three were delivered partly as planned, and two were not delivered as planned: (1) handing out of the welcome folder, which was the responsibility of all health care professionals, was not implemented as planned in either of the two departments. The welcome folder was only handed out a few times in one of the departments, and the importance of activity during hospitalization was not mentioned when handing out the folders; (2) the WALK-plan was partly delivered as planned in both departments. In department X, nurses and sometimes physicians and physiotherapists discussed WALK-plans for all patients at staff conferences, but only when the head nurse was present. Notes about the assigned WALK-plan were put on a staff board displaying all patients admitted to the department. These WALK-plans were to be handed to the patients by either a physician or a nurse (fidelity I) or a physician, a nurse or a physiotherapist (fidelity II), but were not always handed out. In fidelity period I, primarily nurses and physiotherapists discussed WALK-plans, and this discussion behavior spread to all staff and patients in fidelity period II. However, there was inconsistency in the focus put on WALK-plans depending on the amount of busyness in the department. In department Y, WALK-plans were handed out to all patients daily, but only by physiotherapists. Nurses and physicians did not take part in handing out WALK-plans; (3) the WALK-path was partly delivered as planned in both departments. A WALK-path was marked in the hallway in both departments. In department X, some nurses and sometimes physiotherapists introduced patients to the WALK-path and encouraged patients to use the path. Not all patients were seen using the path. In department Y, only physiotherapists introduced patients to the WALK-path and encouraged the patients to use it; (4) in neither of the departments, the posters with exercises were used and therefore not delivered as planned; and (5) the self-service was delivered differently in the two intervention departments. In department X, the self-service on beverages and clothes was not delivered as planned during fidelity period I, since no health care professionals motivated or instructed patients to pick up clothes and beverages. During fidelity period II, the self-service on both beverages and clothes was delivered partly as planned, since patients used the self-service (partly assisted by nurses) although some nurses still served beverages to patients with independent walking ability. In department Y, the self-service on beverages and food was delivered partly as planned, since patients used the self-service (partly assisted by nurses), and some health care professionals encouraged patients to walk to the living room during mealtime.

**Table 5:** Fidelity analysis

	Fidelity period I (Hospital A)	Fidelity period II (Hospital A)	Fidelity period III (Hospital B)
**Welcome folder**	**Adherence:** Handing out of a welcome folder when welcoming a patient to the department was observed once. **Quality of delivery:** During the one-time delivery of a welcome folder, no information was given on WALK-Cph components or the importance of being active during hospitalization. **Participant responsiveness:** Very little engagement was observed, since only one folder was handed out. **Context:** The welcome folder was one of many documents to be handed out to patients on admission. **Fidelity:** This component was not delivered as planned since only one welcome folder was handed out during observations.	**Adherence:** Handing out of a welcome folder when welcoming a patient to the department was observed twice. **Quality of delivery:** During the two sessions, no information was given on WALK-Cph components or the importance of being active during hospitalization. **Participant responsiveness:** Very little engagement was observed, since folders were only handed out twice. **Context:** WALK-logo stickers to be put on welcome folders as reminder for the staff were not used. **Fidelity:** This component was not delivered as planned since only two welcome folders was handed out during observations.	**Adherence:** The WALK-Cph intervention and the importance of being active during hospitalization were not mentioned in the welcome folder. **Quality of delivery:** No observations could inform on welcoming of patients and delivery of this intervention component. **Participant responsiveness:** No observations could inform on participant responsiveness. **Context:** No observations could inform on context. **Fidelity:** This component was not delivered as planned since the WALK-Cph intervention and the importance of being active during hospitalization were not mentioned in the welcome folder and no folders were handed out during observations.
**WALK-plan**	**Adherence:** Prescription of WALK-plans was discussed daily for all patients before physicians’ rounds or during conferences (but only when the head nurse was present). Some, but not all physicians handed out WALK-plans to patients, but mostly when reminded to do so by a nurse. Sometimes physicians forgot to hand out prescribed WALK-plans. Often, nurses handed out WALK-plans on behalf of the physician. Physiotherapists were not seen handing out WALK-plans. WALK-plans were not handed out at discharge. **Quality of delivery:** Physicians forgot to address the WALK-project and WALK-plans at conferences and forgot to hand out WALK-plans at rounds. It was unclear who was responsible for signing discharge WALK-plans and this part of the intervention was forgotten. **Participant responsiveness:** Nurses (primarily the head nurse) and the physiotherapists engaged in and took the initiative to discuss which patients to give WALK-plans. Both nurses and physiotherapists used the staff board for information on assignment of WALK-plans. Physicians did not initiate evaluation of patients regarding WALK-plans. Some physicians showed positive attitudes towards WALK-plans, but still needed reminders on prescribing and handing out WALK-plans whereas others were skeptical about prescribing and handing out WALK-plans, and not all physicians were aware of the WALK-Cph intervention. Physiotherapists were observed using the WALK-plan when training with patients. **Context:** Lack of clarity on who was responsible for handing out WALK-plans. Physicians spent a lot of time in front of computers updating patient journals in new IT-system. Lack of time or busyness kept nurses from handing out WALK-plans. Board meeting is at a time (1 pm) when the physiotherapist is not able to attend the meeting. **Fidelity:** This component was partly delivered as planned, since WALK-plans were discussed, prescribed and handed out, but not by all physicians. During this period adaptions were made to the WALK-plan component. It was decided that WALK-plans could be signed by and handed out by nurses and physiotherapists.	**Adherence:** During the first part of this feasibility period, it was observed that WALK-plans were discussed by nurses and physicians at conferences when the head nurse was present. Further, it was observed that some nurses and some physicians prescribed WALK-plans before or during physicians’ rounds. Prescription of WALK-plans was noted on a board in the nurses’ office. It was observed that WALK-plans were handed out by nurses (most often) and physicians (sometimes). **Quality of delivery:** When the head nurse was present, nurses and physicians discussed WALK-plans during conferences. Both physicians, nurses and nursing assistants talked about WALK-plans. Also, patients were observed talking about WALK-plans. By the end of the period, the head nurse decided that prescription of WALK-plans should not be discussed at the conferences. **Participant responsiveness:** Observations showed that some physicians, but not all, handed out WALK-plans when doing rounds. All physicians were aware of the WALK-Cph intervention. Some nurses were observed reminding physicians to prescribe and hand out WALK-plans. **Context:** The head nurse described that overcrowding and lack of staff due to sick leave influenced how much focus nurses put on WALK-plans and other components. **Fidelity:** This component was partly delivered as planned since observations showed that WALK-plans were discussed at conferences and prescribed during the first part of the period. At the end of the period, overcrowding and lack of staff resulted in lack of focus on the WALK-plans.	**Adherence:** Throughout the feasibility period it was observed that WALK-plans were handed out to patients by physiotherapists. Further, it was seen that physiotherapists evaluated every patient’s need for a WALK-plan on daily basis. At the multidisciplinary conferences, only physiotherapists mentioned WALK-plans. Observations showed that physicians and nurses did not take part in prescribing or handing out WALK-plans. **Quality of delivery:** Daily, physiotherapists evaluated WALK-plan relevance for patients in the department and handed out WALK-plans. Physicians and nurses did not take part in prescribing and handing out WALK-plans. **Participant responsiveness:** It was observed that only physiotherapists took part in prescribing and handing out WALK-plans. **Context:** It was observed that prescription of WALK-plans was noted on a board in the office and that the board and WALK-plan status were updated all weekdays. Observations showed that WALK-plans, when handed out to a patient, could hang on a board next to the patient’s bed. **Fidelity:** This component was delivered partly as planned since physiotherapists systematically assessed patients and handed out WALK-plans, but physicians and nurses did not take part in prescribing and handing out WALK-plans.
**WALK-path**	**Adherence:** Some of the health care professionals were aware of the WALK-path, introduced colleagues to it and used the path to mobilize patients. The first weeks of the feasibility period patients used the WALK-path and some registered number of rounds on a board in the hallway. Some patients were walking together, and some competed on number of rounds. The path was used a lot by a few patients, and others did not use the path. By the end of the period, less activity was seen along the WALK-path. Patients stayed in bed, waiting for the physicians to come by on rounds. **Quality of delivery:** The WALK-path was marked in the hallway as planned, but only some health care professionals motivated patients to use it and introduced colleagues to the WALK-path intervention component. **Participant responsiveness:** Some nurses motivated patients to use the WALK-path and seemed happy about the path. When patients were using the WALK-path some nurses encouraged them to continue. There were no observations on physicians doing so. Some physiotherapists used the WALK-path during training sessions with patients. **Context:** Extra walking aids that should be available for patients needing these as support when walking disappeared during this period (some were found in adjacent ward). Often, food trolleys, chairs and beds were left on the WALK-path (and walking along the part could be an obstacle course). **Fidelity:** This component was delivered partly as planned, since not all health care professionals were engaged in motivating patients to use the WALK-path and be active. Not all patients all patients assigned a WALK-plan were introduced to the path or used the path.	**Adherence:** During this period, more patients were observed using the WALK-path than in the first fidelity period, and a few of them registered number of rounds on a board in the hallway. Some relatives were observed walking along the WALK-path with their hospitalized relative. **Quality of delivery:** The WALK-path was marked in the hallway as planned, and some nurses motivated patients to use it and introduced colleagues to the WALK-path intervention component. **Participant responsiveness:** Some nurses were seen encouraging patients to continue walking when already using the WALK-path. Physicians were not seen encouraging patients to use the WALK-path. Physiotherapists were not observed referring patients to the WALK-path but used it as a part of their training program **Context:** During this period parts of the WALK-path were often blocked by trolleys, old beds etc. Observations showed that during daytime, lights in the bedrooms were dimmed and most patients were lying in their beds. Chairs marked with the WALK-Cph logo on resting areas along the WALK-path disappeared during the period, and no other chairs were available in the hallway or resting areas. Extra walking aids, which should be available for patients needing these as support when walking, were missing during this period. Business is mentioned by some nurses as a barrier for encouraging patients to walk. **Fidelity:** This component was delivered partly as planned, since not all health care professionals were engaged in motivating patients to use the WALK-path and be active. Not all patients all patients assigned a WALK-plan were introduced to the path or used the path.	**Adherence:** Observations showed that the WALK-path was used by patients on a daily basis. Patients were mostly seen walking alone, but sometimes relatives were walking along with their hospitalized relative. Physiotherapists were seen using the WALK-path during testing and training with patients. **Quality of delivery**: The WALK-path was marked in the hallway as planned. The WALK-path was mostly delivered as a unidisciplinary intervention component by physiotherapists. **Participant responsiveness:** Observations showed that physiotherapists reminded patients to use the WALK-path and when meeting patients along the WALK-path they encouraged them to continue. It was seen that nurses and physicians did not refer patients to the WALK-path, but nurses talked to patients about the importance of being active when hospitalized. **Context:** It was observed that chairs marked with the WALK-Cph logo disappeared from the resting areas, but other chairs were available along the WALK-path. **Fidelity:** This component was delivered partly as planned, since physiotherapists referred patients to the WALK-path, but physicians and nurses did not take part in promoting the WALK-path.
**Posters**	**Adherence:** The posters were not put up in time and therefore not used. **Quality of delivery**: No observations could inform on quality of delivery. **Participant responsiveness:** No observations could inform on participant responsiveness. **Context:** No observations could inform on context. **Fidelity:** The posters were not put up and therefore not delivered as planned.	**Adherence:** Once, a nurse was observed telling a patient to perform one of the exercises from the poster. **Quality of delivery**: Only one time we observed that the posters were used as planned. Therefore, not enough observations could inform on quality of delivery. **Participant responsiveness:** No observations could inform on participant responsiveness. **Context:** No observations could inform on context. **Fidelity:** The posters were not used and therefore not delivered as planned.	**Adherence:** Twice, patients were seen using the posters. **Quality of delivery:** Two times, a patient was instructed to the exercises on the posters. **Participant responsiveness**: Two times, physiotherapists were seen giving patients instructions on using the posters. **Context:** Chairs to use when performing exercises, e.g. chair-stand or heel raises, were placed next to the posters. **Fidelity:** Only a few times, patients were instructed to use the posters and therefore this component was not delivered as planned.
**Self-service on clothes and beverages**	**Adherence:** Throughout the period, patients were seen using the self-service on beverage. During the whole period, nurses (and students) were observed bringing food and beverages to patients, who were able to walk independently, and to patients prescribed with a WALK-plan. Few patients were seen picking up clothes, and only twice nurses were seen giving patients instructions on where to pick up clothes. **Quality of delivery:** Not all patients were informed on where to pick-up clothes and beverages. Patients were encouraged to pick up food and beverages by some nurses, yet some nurses brought food and beverages to patients who were able to walk independently. **Participant responsiveness:** Some, but not all, health care professionals were seen motivating patients to pick up clothes and beverages **Context:** Throughout the period, beverages were available in the hallways. Some patients were not given information on where to pick up clothes. **Fidelity:** The self-service on beverages was not delivered as planned. Patients were using the self-service on beverages themselves, but nurses served beverages to patients. The self-service on clothes component was not delivered as planned.	**Adherence:** Many patients were observed picking up beverages, some when they were walking rounds along the walking path. Some nurses were seen serving beverages to patients with WALK-plans or patients who were able to walk independently. It was observed that nurses served beverages to patients when serving meals. Patients were mostly sitting on or lying in their beds during meals. Patients were observed picking up clothes and some asked where they could pick up clothes. **Quality of delivery:** Patients were encouraged to pick up beverages. Some nurses instructed patients on where to pick up clothes. When asked other nurses showed patients where to pick up clothes **Participant responsiveness:** Some nurses encouraged patients to pick up beverages and some nurses continued to serve beverages to patients who were able to walk. Some nurses and nursing assistants referred patients to pick up clothes themselves. **Context:** Throughout the period, beverages were available in the hallways. **Fidelity:** The self-service on beverages was partly delivered as planned since many patients picked up beverages themselves, but some nurses continued to serve beverages to patients who were able to walk. The self-service on clothes was partly delivered as planned since some nurses, when asked, encouraged patients to use it.	Pick up of clothes was not possible due to rules at this department. **Adherence**: Throughout the period most of the department’s patients were using the self-service on beverages and on a daily basis, patients were seen using the living room to eat their meals, watch television, and talk to relatives or other patients. **Quality of delivery:** Only nurses were seen delivering this component, and only some nurses encouraged patients to pick up food and beverages by themselves. **Participant responsiveness**: It was observed that some nurses encouraged patients to walk to the living room to eat their meals there, and some nurses did not. Some nurses were observed serving meals in the bedroom to patients who were able to walk. Physiotherapists were not seen encouraging patients to walk to the living room to eat the meals. No observations showed physicians engaging in getting patients to walk to get food or beverages. **Context:** Throughout the period, beverages were available in the hallway and the living room, and during mealtime meals were available in the living room. **Fidelity:** This component was delivered partly as planned, since observations showed that many patients were encouraged to pick up food and beverages and did so, but not by all staff.

## Discussion

In this study, we investigated the feasibility and the implementation fidelity of the co-designed WALK-Cph intervention. With regard to feasibility, the recruitment procedures, assessment procedures, and the method for assessment of 24-h mobility were all found to be feasible. Concerning fidelity, we observed that three of the five WALK-Cph intervention components were only partly implemented as planned during the fidelity periods (the WALK-plans, the WALK-path, and self-service on beverages) and none of the components was implemented as planned.

The inclusion of patients from the two intervention departments was successful. During the three 4-week feasibility periods, two thirds of the eligible patients were willing to participate. This level of recruitment is similar to previous cohort studies of older medical patients conducted in our research unit [1, 41]. All included patients completed the assessments and accepted to wear the *activ*PAL3^TM^ activity monitors. Therefore, based on the feasibility results, it was concluded that further evaluation of the effectiveness of the intervention in a randomized controlled trial could be carried out.

The activity assessment showed that the patients were inactive for approximately 22 h per day despite being able to walk without help. Similar inactivity levels have been found in previous studies in older hospitalized patients across countries and health care systems [1, 3, 4]. This finding underlines the relevance of supporting the full implementation of projects like WALK-Cph [36] to achieve increased mobility among older medical patients.

Recently, recommendations for physical activity and sedentary behavior for older adults during hospitalization for an acute medical illness were published based on a DELPHI study [67]. The recommendations stressed the importance of acting with respect for individual patient capabilities, incorporating physical activity throughout daily care, sharing the responsibility, and involving patients and stakeholders to facilitate implementation. This view is supported by others in a recent scoping review [68] and meta-synthesis [69], both of which state that promotion of mobility in hospitalized older adults is a team effort and requires clarity around interprofessional responsibilities, as well as applying a systems approach that aligns patients’ mobility expectations with those of caregivers [68, 69]. Also, according to Bowen et al. [40], it is important to understand the perspectives of stakeholders who will affect and be affected by the intervention and who are important if the intervention is to be implemented into practice. This also includes hospital management and backup support who are vital for successful implementation [70]. Despite the WALK-Cph intervention being co-designed, our observations confirmed that the intervention still lacked clarity with regard to interprofessional responsibilities and the responsibility for ensuring patient mobility [22].

During the initial phase of implementing the WALK-Cph intervention, two of the five WALK-Cph intervention components to promote in-hospital mobility were not implemented as planned. Not all health care professionals assumed responsibility for promoting mobility. However, there were smaller groups of health care professionals at the two intervention departments who prescribed WALK-plans and motivated patients to be active and to use the WALK-path and self-service during hospitalization. These health care professionals may be viewed as innovators and early adopters as described in Rogers’ diffusion of innovation theory [71–73], which suggests that such individuals adopt new ideas before they spread within a social system (e.g., a hospital department) and are adopted by the majority [71–73]. At this point, however, it is unknown whether the WALK-Cph intervention components will be adopted by all health care professionals at the two intervention departments [73]. Nevertheless, since three components were at least partly implemented as planned, and, specifically, the two core components were implemented partly as planned, it was decided to proceed with further investigation of the effectiveness and the implementation of the WALK-Cph intervention.

According to Carroll [63] and Hasson [62], interventions described in detail are more likely to be implemented with high fidelity than interventions with vague descriptions. Detailed descriptions include more facilitation strategies to support the intervention deliverers (e.g., manuals, guidelines, training, and feedback), which may enhance, i.e., quality of delivery [62]. The WALK-Cph components were described in detail regarding the amount of physical activity. In some cases, responsibility for delivery of the components was described in more general terms, i.e., at professional levels without indicating main responsibilities (i.e., that all health care professionals are to motivate the patients to use the WALK-path). However, while the intervention was designed to be simple, it may have been perceived as complex by the health care professionals due to interacting components, different behaviors required by those delivering the intervention, and the interdisciplinary teamwork required [32, 74]. Perceived complexity is one of the attributes in Rogers’ diffusion of innovation theory, which posits that innovations, e.g., new interventions, that have low perceived complexity are more likely to succeed [71]. Complex interventions have more possibility for variation in delivery, and thus, more risk of not being implemented as intended [62]. It is important to note that Rogers’ attributes relate to subjective perceptions of various aspects of innovations. This means that some people may find a new intervention basic and simple, whereas others may consider the same intervention complex and challenging.

The WALK-Cph intervention was developed in a co-design process to engage stakeholders and to adapt the intervention to the local context. We hoped that this approach would increase the likelihood of successful implementation [75, 76]. However, we found limited implementation fidelity of the intervention because some components were not implemented. According to Boyd [77], the involvement and active participation of health care professionals is fundamental in co-design work. For various reasons, not all health care professionals in the departments participated in the co-design process. It seems likely that those who participated in the workshops were amongst the early adopters [71] and that the remaining health care professionals might need more time to recognize the utility and advantages of the intervention to fully endorse it. Implementing change, such as introducing a new intervention in a clinical setting, can generate different responses from the health care professionals who are expected to use or carry out the intervention [78]. Even though the WALK-Cph intervention was developed in a co-design process, the perception of the intervention differed between health care professionals, which is consistent with Rogers’ perceptions of innovation attributes. It is the individuals’ perceptions of the attributes of an innovation that affect its adoption, not the attributes classified “objectively” by experts or researchers [71].

Only one physician participated in the workshops, which might imply a resistance toward the intervention. To investigate if this was the case, we carried out barrier screening interviews with physicians from the two intervention departments [22] and found that the physicians were reluctant to promote mobility, which included both patient-, context,- and professional factors. The physicians were aware of the necessity and relevance of focusing on in-hospital mobility but believed that nurses should be responsible for mobility. The physicians’ involvement was brought into focus during the co-design workshops, as all groups of stakeholders emphasized the importance of physicians prescribing WALK-plans. The relevance of physicians’ advice on physical activity in older adults is supported by studies reporting that older adults find physician advice important [79–81] and trust physicians the most to deliver health information [82]. However, in our study, it was the physiotherapists and nurses who handed out WALK-plans to the patients and the prescription task. It was planned to be the physicians’ responsibility but was changed to be carried out by other health care professionals. Historically, the roles of nurses and physicians differ, with nurses focusing on patient health and wellbeing and physicians being responsible for diagnoses and medical conditions [83]. These well-established professional roles may in some cases hinder cross-professional collaboration [84], making it difficult to successfully implement interventions such as the WALK-Cph intervention.

We carried out observations of the daily clinical practice based on the assumption that ethnography is inherently contextual, which emphasizes the importance of context in understanding events and meanings [58]. Ethnography describes what people say and do, and their relationship with others, and has proven useful for understanding collective and non-rational dimensions of organizations. Thus, observations provide a picture of the interplay between an intervention and the setting in which it is implemented [58]. We evaluated both the feasibility of conducting a randomized controlled trial in the setting of interest [34] and the fidelity of the intervention over 4 weeks to ensure sufficient time to evaluate if delivery of the intervention adhered to the described intervention and, thus, provided a basis for the intervention to be effective [29]. Since the observations showed that the core components were implemented partly as planned, it was deemed relevant to carry on with efficacy testing of the intervention.

### Limitations

The study has some limitations to consider when interpreting the findings. Firstly, different implementation strategies can be used to support implementation such as guidelines, training, and feedback [62]. After designing the intervention, the departments had the sole responsibility for the implementation of the intervention and, thus, for strategies to enhance implementation. Secondly, during our observations and interviews with physicians from the two departments [22], it was clear that the departments were characterized by a busy schedule and it is therefore likely that putting aside resources to support the implementation of the intervention was not prioritized. Also, lack of time and resources may have overshadowed the observability of the benefits of the intervention, a factor believed to ease the adoption of an intervention [85]. Thirdly, we were not present continuously for 4 weeks and were therefore not able to get a complete picture of adherence and thus of implementation fidelity. However, it is rarely feasible to assess all features of implementation fidelity why a selection based on practical circumstances can be necessary [29]. Fourthly, feasibility was only assessed during hospitalization even though the following randomized controlled trial was designed for assessments on admission and 4 weeks after discharge. Therefore, we cannot conclude on the feasibility of conducting assessments in the patients’ homes after discharge. In previous feasibility and randomized controlled trials, however, we have conducted at-home assessments without major obstacles [41, 86], and we therefore considered the hospital setting most important for the present study.

## Conclusions

The co-designed WALK-Cph intervention was deemed feasible concerning the recruitment and assessment procedures for conducting a randomized controlled trial to investigate the effectiveness of the intervention. The intervention was not implemented with fidelity. However, as the two WALK-Cph intervention components that were pre-defined as core (the WALK-plans and the WALK-path) were partly implemented as planned, it was decided to continue with further testing of the WALK-Cph intervention in a large-scale trial.

## Supplementary Information

**Additional file 1.** The WALK-Cph intervention components.

## Data Availability

The data contain potentially identifying or sensitive information that could compromise the privacy of the participants or those being observed and are therefore not publicly available according to regulations set out by the Danish Data Protection Agency, but are available from the corresponding author on reasonable request.

## Abbreviations

WALK-Cph: The WALK-Copenhagen project
DEMMI: The De Morton Mobility Index
LSA: The Life-Space Assessment
ADL: Activities of daily living
OMC: The Orientation-Memory-Concentration test
*activ*PAL: The *activ*PAL3^TM^ accelerometer
