# Supplementary material for: Feasibility and implementation fidelity of a co-designed intervention to promote in-hospital mobility among older medical patients—the WALK-Copenhagen project (WALK-Cph)
Source: Pilot Feasibility Stud. 2022 Apr 9;8:80. doi: 10.1186/s40814-022-01033-z (PMC8994315; doi:10.1186/s40814-022-01033-z)
Supplement: Supplementary file 1 — Additional file 1. The WALK-Cph intervention components. [file 40814_2022_1033_MOESM1_ESM.docx]

**Additional file 1: The WALK-Cph intervention components**

| **COMPONENTS** | **INTERVENTION** | **DESIGN** |  |
| --- | --- | --- | --- |
| **During hospitalization** | | | |
| Welcome folder | On admission, health care professionals hand out a welcome folder, when introducing patients to the departments. When handing out the welcome folder, health care professionals emphasize the importance of walking during and after hospitalization. | A green WALK-Cph logo is printed on the front of the welcome folder.  The welcome folder contains a paragraph describing the importance of walking during and after hospitalization. |  |
| WALK-plan | Daily, physiotherapists and nurses evaluate all patients and decide: 1) who to prescribe a WALK-plan; 2) level of the WALK-plan; 3) changes to current WALK-plans. All WALK-plans are noted on a patient board in the common office. The physicians prescribe WALK-plans and motivate all patients to walk. The WALK-plans are handed out to the patients by either the nurse or the physiotherapist.  All groups of health care professionals are responsible for motivating the patients to walk and follow the WALK-plans. | Three different WALK-plans with WALK-Cph logos are preprinted  Level 1 (red): 1 minute – three times a day  Level 2 (yellow): 5 minutes – three times a day  Level 3 (green): 10 minutes – three times a day + suggestion for three exercises identical to the exercises on the posters. |  |
| WALK-path | Daily, the patients are motivated by all health care professionals to use the walk path for walking and exercising. Introduction and assistance are provided by nurses, nursing assistants or physiotherapists until the patients are self-reliant. The patients should exercise by the walk path at least once a day. | A green line is stuck on the floor the whole length of the department and is marked with ’WALK-path’ and green feet. A green chair and a poster with exercises is placed at each common area (rest area). A small whiteboard is placed in the common area for the patients to mark the number of rounds or minutes they have walked. Daily, the night staff update the board (count the number of rounds). |  |
| Posters with exercises | Posters with three exercises are placed at the rest areas and in the patient rooms All health care professionals motivate the patients to seek inspiration from the posters and perform the exercises. | The posters show three exercises:   1. Walking: Walk by the WALK-path 2. Balance: Stand on one leg at a time (with a chair for aid) 3. Strength: Stand up from a chair and sit down |  |
| Self-service on clothes* and beverages | The patients collect clothes from the wardrobes. On admission, the health care staff introduce the patients to the wardrobes and motivate the patients to collect clothes.  The patients collect beverages from the beverage wagon and the refrigerator. On admission, the health care staff introduce the patients to optimal hygiene, the beverage wagon and the refrigerator and motivate the patients to collect beverages. | The wardrobes, from which the patients may collect clothes, are marked with WALK logos. |  |
| **After discharge (one component)** | | | |
| Patients with a WALK-plan, who are discharged with a rehabilitation plan, will be contacted by phone by a municipal therapist | At discharge, the physiotherapist makes sure to note in the rehabilitation plan that the patient is discharged with a WALK-plan. One to five days after discharge, a therapist from the municipality contacts the patient by phone to motivate the patient to continue to follow the WALK-plan. |  |  |
| Patients with a WALK-plan, who are discharged without a rehabilitation plan but receive home care, will be contacted by phone by home health care personnel | At discharge, the nurse makes a note for the municipality in the discharge papers stating that the patient is discharged with a WALK-plan.  After discharge, when visiting the patient, the home care health care personnel motivate the patient to follow their WALK-plan. |  |  |

**This component was not a part of the intervention in Department Y*
